# Ubiquitination in melanoma pathogenesis and treatment

**DOI:** 10.1002/cam4.1069

**Published:** 2017-05-23

**Authors:** Jinyuan Ma, Weinan Guo, Chunying Li

**Affiliations:** ^1^ Department of DermatologyXijing hospitalFourth Military Medical UniversityXi'anShaanxiChina

**Keywords:** Deubiquitination, melanoma, pathogenesis, treatment, ubiquitination

## Abstract

Melanoma is one of the most aggressive skin cancers with fiercely increasing incidence and mortality. Since the progressive understanding of the mutational landscape and immunologic pathogenic factors in melanoma, the targeted therapy and immunotherapy have been recently established and gained unprecedented improvements for melanoma treatment. However, the prognosis of melanoma patients remains unoptimistic mainly due to the resistance and nonresponse to current available drugs. Ubiquitination is a posttranslational modification which plays crucial roles in diverse cellular biological activities and participates in the pathogenesis of various cancers, including melanoma. Through the regulation of multiple tumor promoters and suppressors, ubiquitination is emerging as the key contributor and therefore a potential therapeutic target for melanoma. Herein, we summarize the current understanding of ubiquitination in melanoma, from mechanistic insights to clinical progress, and discuss the prospect of ubiquitination modification in melanoma treatment.

## Introduction

Melanoma is among the most aggressive cancers arising from the malignant transformation of melanocytes 1. In 2015, there was an estimation of 73,870 new cases and almost 10,000 deaths of melanoma in the United States, accounting for nearly 75% of all skin cancer deaths 2. Despite the current breakthroughs in targeted therapies and immunotherapies, as well as the advances in early diagnosis and prevention, the prognosis of melanoma remains unoptimistic, especially for those disseminated to distant sites and visceral organs (American Joint Committee on Cancer (AJCC) stage IV), with a median survival time of only 6–9 months and a 3‐year survival <20% 3, 4, 5.

Since the advent of high‐throughput sequencing technologies and bioinformatics in the recent years, the mutational landscape and molecular pathogenetic basis for melanoma have been gradually identified 6, 7, 8, 9, 10. The most common genetic targets, *BRAF* and *NRAS*, are frequently mutated in nearly 50% and 20% melanomas, respectively 11, which result in hyperactivation of MAPK and PI3K pathways and subsequent uncontrolled proliferation of melanoma cells 6, 12. In addition, genetic variations of key cell cycle regulators and transcriptional factors, such as CDKN2A and microphthalmia‐associated transcription factor (MITF), have also greatly contributed to melanoma carcinogenesis 13, 14, 15. These discoveries opened new avenues for potential targeted therapeutic strategies, and the specific BRAF inhibitor, vemurafenib, initially demonstrated dramatic efficacy followed by other competitive BRAF and MEK inhibitors 16, 17, 18, 19. Then the combination of BRAF inhibitor and MEK inhibitor (always consists of dabrafenib and trametinib) rapidly became the standard targeted treatment for BRAF^V600E^ mutation‐positive melanoma.^20^.Aside from these, the immune checkpoint inhibition approach has also gained unprecedented progress for melanoma management and even leads the way for other malignancies 21, 22, 23. However, responses of these therapies are restricted to a subgroup of melanoma patients, and the disease relapse will inevitably occur due to the transient durability, strikingly limiting the prolongation of patients' survival 18, 19, 21. Therefore, identifying alternative pathogenetic mechanisms and novel treatment strategies remains an active area of research for improving the outcome of melanoma patients.

Ubiquitination is a protein posttranslational modification reservedly regulated by a series of ubiquitination‐associated enzymes 24. The cellular functions of ubiquitination span a wide spectrum that includes cell death, DNA damage repair, autophagy, proteasomal degradation of proteins and metabolism 25. Thus, dysregulation of ubiquitination has broad consequences that may lead to aberration of tumor‐promoting pathways and tumor‐suppressing pathways. Recently, accumulative evidence has established the critical role of ubiquitination in cancer pathogenesis and therefore revealed the great therapeutic potential of targeting ubiquitination in various cancers 26, 27, 28, 29. This review will summarize the current understanding of ubiquitination in melanoma, from mechanistic insights to clinical progress, and discuss the prospect of ubiquitination modification in melanoma treatment.

## The Biology of Ubiquitination

Ubiquitination is among the most evolutionarily conserved protein posttranslational modification. Ubiquitin is a 76‐amino acid protein that covalently couples to lysine residues on target proteins, with a single ubiquitin conjugated to each lysine called monoubiquitination and ubiquitin chains conjugated to the lysine residue called polyubiquitination 30. The bond of ubiquitin to the target protein is mediated by a cascade of enzymes, namely, the ubiquitin‐activating enzyme (E1), the ubiquitin‐conjugating enzyme (E2), and the ubiquitin ligase (E3). Specifically, ubiquitin is first activated by E1 in an ATP‐dependent manner, and then transferred onto E2 and in conjunction with E3, which is considered to be the most important in determining ubiquitination specificity, recognizing a substrate and mediating isopeptide bond formation between the C‐terminus of ubiquitin and substrate lysine 31, 32. In line with other posttranslational modification, the process of ubiquitination is reversible, with the removal of ubiquitin from substrates regulated by deubiquitinating enzymes (DUBs) 33. Up to date, more than 600 annotated E3 ubiquitin ligase and 100 deubiquitinating enzymes have been identified, forming a molecular network governing intracellular ubiquitination dynamics 25.

**Figure 1:**
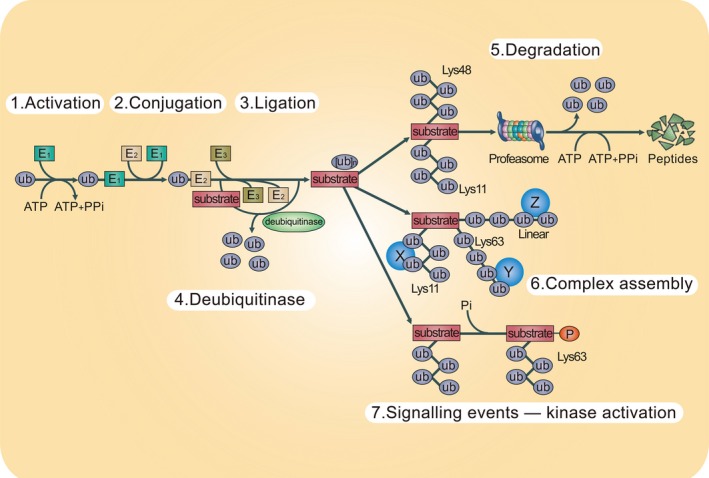
The UPS system. The ubiquitination system contains a series of reactions performed by the enzymes of the UPS. As the beginning of the reaction, the ubiquitin is transferred to E1 enzyme in an ATP‐dependent manner (step 1). Then the activated ubiquitin is transferred and conjugated to an E2 enzyme (step 2). Subsequently, the E2 enzyme carries the ubiquitin to the E3 enzyme (step 3). The E3 that mediates substrate specificity can covalently ligate ubiquitin to the substrate protein. The process may be repeated to form a polyubiquitin chain on the target protein. Deubiquitination is a reverse reaction of ubiquitination (step 4). Ligation of polyubiquitin has various consequences on the recipient protein. For example, Lys11‐ and Lys48‐linked polyubiquitin chains can target substrate proteins and lead to the proteasomal degradation (step 5). Conversely, Lys63‐ and Lys11‐linked chains promote to form certain signaling complexes (step 6). Moreover, Lys63‐linked chains can participate in the cell signaling events such as facilitating the proteins phosphorylation by its kinase (step 7). X, Y, and Z indicate ubiquitin‐binding proteins. Pi, inorganic phosphate; PPi, inorganic diphosphate; Ub, ubiquitin. UPS, ubiquitin–proteasome system.

The biological function of ubiquitination is far beyond its classic proteolytic role in tagging proteins for degradation. Depending on the modification type and the specificity of ubiquitinated substrates, ubiquitination participate in a wide range of processes, including protein localization 34, 35; assembly of multiprotein complexes^36^, ^37^; metabolic modification 38; inflammatory signaling 39; autophagy 40; DNA damage response, and regulation of enzymatic activity (Fig. 1) 24. Therefore, dysregulated deubiquitination can lead to improper protein localization and protein–protein interactions, intracellular metabolic disorder, accumulation of misfolded proteins, amplification of inflammatory response, as well as aberrant activation of enzymes and signaling pathways, which are detrimental to the cellular homeostasis and greatly involve in the pathogenesis of many diseases 26, 41.

Notably, there are subsets of proteins termed ubiquitin‐like proteins (UBLs), which are functionally or structurally similar to ubiquitin, including NEDD8, SUMO, ISG15, and FAT10. These proteins can be conjugated to target proteins by an enzyme cascade analogous to ubiquitin E1, E2, and E3. To be specific, SUMOylation uses AOS1 or Uba2 as E1 and Ubc9 as E2, and NEDDylation usually uses APPBP1 or Uba3 (E1‐like) and Ubc12 (E2‐like). Generally, UBL protein modifications are not associated with protein degradation, but contribute to protein subcellular localization, transportation and protein–protein interactions 33, 34, 35.

## Proteomic Techniques to Identify the Ubiquitination Events

In order to investigate the role of ubiquitination in different diseases including melanoma, it is important to identify protein ubiquitination sites and the related ubiquitination events. However, it is very challenging due to the rather low abundance of ubiquitinated proteins under normal physiological conditions and the few lysine residues modified in an ubiquitinated protein 42, 43. Conventionally, the precise site of ubiquitination for a single protein is revealed by mutagenesis of the putative target residues from lysine into arginine, which abolished the capacity to be ubiquitinated, and the subsequent detection is usually achieved through immunoblotting analysis 44, 45. Later on, the mass spectrometry (MS)‐based proteomic approach has been developed to enhance the efficiency for the identification of ubiquitination sites under physiological conditions. The signature peptide adducts derived from ubiquitin can be detected of a mass shift of 114.043 Da by MS 46. Because of the low abundance of ubiquitinated proteins in cells, it is necessary to enrich for the ubiquitinated proteins for successful identification by MS. Recently, several approaches have been used for the isolation of ubiquitinated proteins. The first is the small affinity tags for labeling ubiquitin with the most common one as His_6_ 47. These short peptide tags are engineered at the N‐terminus of ubiquitin and the tagged ubiquitin is expressed in cells. Once a protein is ubiquitinated, it is also labeled by the affinity tag on the ubiquitin. Therefore, tagged ubiquitinated proteins can be isolated using commercially available resins (Ni‐NTA or TALON for His_6_). The second is the use of high‐affinity anti‐ubiquitin antibodies for isolating ubiquitinated proteins. Several types of anti‐ubiquitin antibodies, including P4D1, FK2, and FK1, are available for the detection of free ubiquitin and ubiquitin conjugation 48, 49. More importantly, the development of several linkage‐specific monoclonal antibodies makes it rather convenient for the isolation of ubiquitinated proteins with specific chain linkages, such as K11‐, K48‐, and K63‐linkages 50. The third approach for isolating ubiquitinated proteins for MS identification is the use of ubiquitin‐binding domains (UBDs). UBDs are small structural entities that have affinity to ubiquitin and over 20 different UBDs have been discovered, such as ubiquitin‐associating domain (UBA) and ubiquitin‐interacting motif (UIM) 51. The ubiquitinated proteins are isolated via the interaction between the UBDs and polyubiquitin chains. Recently, many ubiquitinated proteins and their ubiquitination sites have been identified through this way 52, 53, indicating its great potential in the future.

As mentioned above, the identification of ubiquitin remnant‐containing peptides by MS has been widely used for uncovering ubiquitination events at the proteome level. Nevertheless, for any ubiquitinated protein, the majority of the peptides detected on MS will not contain modified lysine residues, which remarkably reduces the efficiency of the identification 54. Thus, the enrichment of ubiquitin remnant‐containing peptides rather than the ubiquitinated proteins can be more helpful. In 2010, Xu et al. firstly use anti‐diglycyl lysine antibodies to perform ubiquitin remnant profiling 55. Specifically, proteins are extracted from cell lysates and digested with trypsin. Ubiquitin remnant‐containing peptides are enriched by immunoprecipitation with anti‐diglycyl lysine, since the diglycine remnant is an appealing epitope for reorganization of ubiquitinated proteins. Subsequently, this approach is used by numerous other groups and more than 20,000 ubiquitination sites in mammalian cell lines have been uncovered through this way 56, 57, which greatly improves the efficiency of the identification of ubiquitination events at a proteome‐wide level.

## Ubiquitination in Melanoma Pathogenesis

### Mutations of ubiquitination‐related enzymes in melanoma tumorigenesis

Melanoma is a heterogeneous tumor with high mutational load and complex signaling networks 1, 58. In addition to the already known driver mutations, the genetic variations of ubiquitination‐related enzymes uncovered by high‐throughput sequencing are also greatly implicated in melanoma tumorigenesis, with BRCA1‐associated protein‐1 (*BAP1*), F‐box and WD repeat domain‐containing 7 (*FBXW7*) and *PARK2* as the best representations.

*BAP1* encodes a nuclear ubiquitin carboxy‐terminal hydrolase (UCH), one of several classes of deubiquitinating enzymes 59, 60. The mutations of *BAP1* have been first reported in a small number of breast and lung cancer samples, and recently implicated in the pathogenesis of melanoma 60, 61. In 2010, Harbour et al. discovered that the inactivating somatic mutations of *BAP1* were frequently identified in 84% metastasizing uveal melanomas, including 15 mutations causing premature protein termination, and six affecting its ubiquitin UCH domains, which were associated with the significant decrease in *BAP1* mRNA level 62. The knockdown of *BAP1* gene in melanoma cell harboring undetectable *BAP1* mutation developed a rounded epithelioid morphology and grew as multicellular nonadherent spheroids, paralleled with the gene expression profile shifting to a metastasizing pattern. This study evidently implicates mutational inactivation of *BAP1* in the acquisition of metastatic competence in uveal melanoma. Later on, the germline mutations of *BAP1* have been linked to a novel autosomal dominant syndrome characterized by a high penetrance of melanocytic neoplasms. More importantly, some affected patients developed uveal or cutaneous melanomas, demonstrating the role of *BAP1* mutations in conferring increased melanoma risk 63.Therefore, the mutations of *BAP1* contribute to not only melanoma tumorigenesis, but also melanoma metastasis. Furthermore, it has been revealed that low BAP1 expression exhibited a worse survival than those with high BAP1 levels 64. Taken together, the deubiquitinase BAP1 could be considered as a promising prognostic biomarker in melanoma.

Aside from *BAP1*, the genetic mutation of another ubiquitin ligase *FBXW7* has also contributed to the tumorigenesis of melanoma. The FBXW7 protein comprises three functionally critical domains—the dimerization domain, the F‐box domain that allows it to bind and interact with the SCF complex, and the WD40 domain that recognizes a specific consensus, phosphodegron motif within the substrate 65, 66, 67. In a previous study, the inactivated mutations of *FBXW7* were reported to occur in 8.1% melanoma patients, with the majority of these mutations in its WD40 domain that disrupts substrate binding and lead to sustained activation of its substrate oncoproteins 68. Notch1, one of the most canonical substrates of FBXW7 69, was remarkably accumulated in cells upon the loss of FBXW7, and then promoted tumor growth and angiogenesis of melanoma. Notch1 is gradually cleaved by a series of proteolytic process, and the SCF^Fbw7^ ubiquitylates transcriptionally active Notch1 intracellular domain (NICD) 70. Notch1 inhibition could potently prevent inactive FBXW7‐induced melanoma tumorigenesis, rendering Notch signaling as a promising therapeutic target in the subset of melanoma patients harboring *FBXW7* mutations 45. Moreover, the nuclear staining of FBXW7 was in a strong negative correlation with patients' outcome, and forward multivariate Cox regression analysis revealed that nuclear FBXW7 expression was an independent factor for predicting melanoma prognosis. Furthermore, knockdown of FBXW7 showed minimal impact on melanoma cell proliferation, but markedly potentiated the migratory capacity. Therefore, the inactivation of FBXW7 participates in both the tumorigenesis and progression of melanoma 71.

Previous epidemiological studies revealed that melanoma incidence is higher in patients affected by Parkinson's disease (PD) and vice versa, but the underlying mechanism is elusive 72, 73, 74. *PARK2*, the frequently mutated gene in young‐onset PD encoding the E3 ubiquitin ligase Parkin, has been recently implicated in the tumorigenesis of melanoma. An in depth *PARK2* gene dosage analysis and sequencing revealed that germline *PARK2* mutations were present in 25 cases out of 512 melanoma patients, but only 4 in 562 healthy controls. Through the odds ratio (OR) calculations, the putative PARK2‐inactivating variants (including splicing, frameshift, CNVs, and predicted deleterious missense mutations) were strongly associated with melanoma risk when compared with control groups (OR = 3.95, 95% confidence interval = 1.34–15.75). In addition, most of the *PARK2* germline alterations were heterozygous in these melanoma patients, suggesting that one mutated *PARK2* allele is sufficient to modulate melanoma risk 75. Interestingly, the expression level of Parkin in various melanoma cell lines was frequently decreased in comparison to that in normal human melanocytes. Overexpression of Parkin in melanoma cells resulted in cell proliferation inhibition and cell apoptosis of melanoma cells, supporting Parkin as a potent melanoma suppressor 76. To date, the downstream mechanism responsible for the suppressive effect of Parkin in melanoma remains unclear. The expressions of cyclin D1 and E, which are canonical substrates of Parkin, are not significantly associated with the expression of Parkin. In addition, the phosphorylation of Rb was not affected by Parkin inhibition, indicating that alternative signaling pathways may be involved in the role of Parkin. Given that Parkin plays an essential role in maintaining mitochondrial integrity and the changes in mitochondrial dynamics and structure that happen during malignant transformation, it may possibly exert tumor suppressor function through mitochondria. Additional experiments are needed to illustrate this important point 75.

## Ubiquitin Modification of Proteins in Key Signaling Pathways in Melanoma Pathogenesis

### NF‐κB pathway regulated by ubiquitination

**Figure 2:**
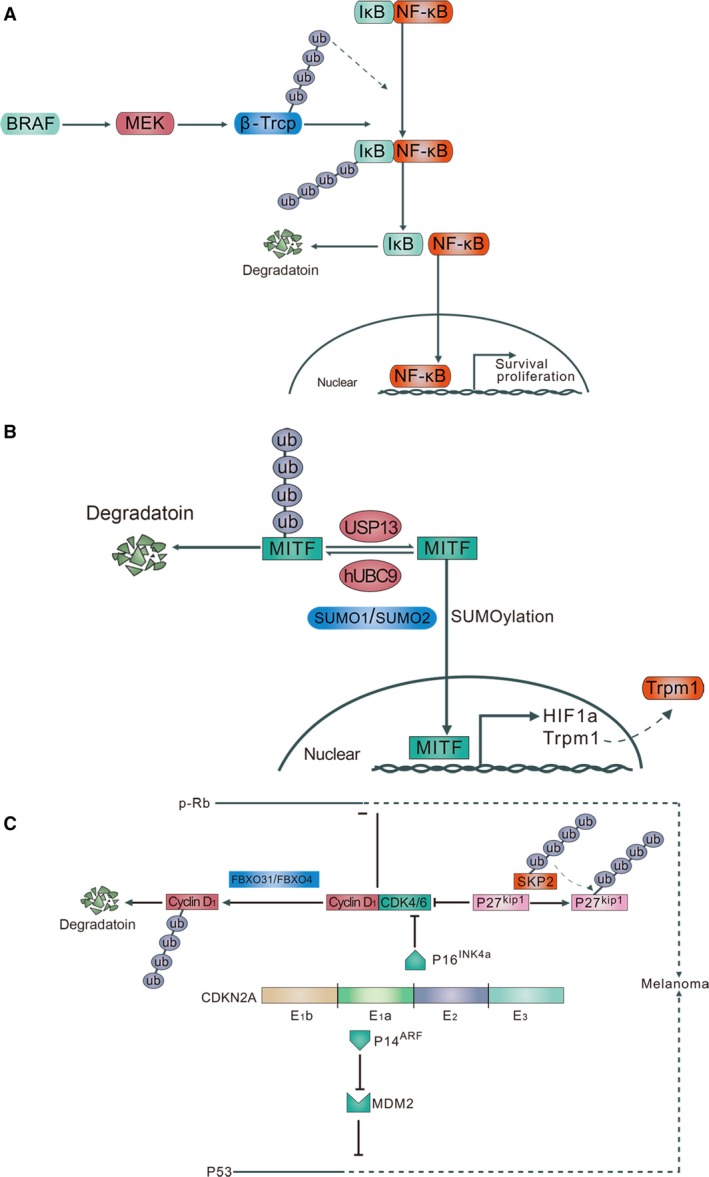
(A) Ubiquitination mediates the NF‐*κ*B activation and nuclear translocation. *β*‐Trcp is the main E3 ligase in the I*κ*B ubiquitination reaction. It can connect the ubiquitin chains to the I*κ*B, facilitating the I*κ*B phosphorylation, which subsequently leads to the nuclear translocation of NF‐*κ*B. In addition, Braf promotes the expression of *β*‐Trcp. (B) Ubiquitination and deubiquitination of MITF. The ubiquitin‐conjugating enzyme hUBC9 directly interacted with MITF and subjected it for degradation by ubiquitin‐proteasome system. However, deubiquitinase USP13 can stabilize MITF expression and prolong its half‐life. Additionally, SUMOylation of MITF by SUMO1/SUMO2 mediates MITF binding to the HIF1A promoter and increases its transcriptional activity. (C) Ubiquitination of proteins in cell cycle.*CDKN2A* encodes two melanoma inhibitors: P14^ARF^ and P16^INK^ ^4a^, both of which are partly coded by the exon 2 and exon 3 regions of *CDKN2A*, and down‐regulates MDM2 (P53 inhibitor) and CDK4/6‐cyclin D1 pathway (suppresses the Rb protein expression), respectively. However, FBXO31/FBXO4 leads to G1 arrest through reducing cyclin D1 expression, subsequently impedes tumor cell growth. On the other side, SKP2 mediates CDK inhibitor P27^kip1^ ubiquitination and degradation, and promotes cell cycle in melanoma. Ub indicates ubiquitin; P, inorganic phosphate; E1a/E1b/E2/E3, exons on *CDKN2A*. MITF, microphthalmia‐associated transcription factor.

NF‐*κ*B is a prosurvival factor which governs the transcription of multiple genes involved in growth, inflammation, and anti‐apoptosis 77, 78. The transcriptional activity of NF‐*κ*B is inhibited by I*κ*B, which binds to NF‐*κ*B and sequesters it in the cytosol. In order for NF‐*κ*B to be activated, I*κ*B is subjected to phosphorylation followed by ubiquitination‐dependent degradation which leads to the nuclear translocation of NF‐*κ*B and thereafter activates its target genes 79, 80. As reported previously, NF‐*κ*B is constitutively activated at high levels in melanoma cells compared with melanocytes, which results from the dysregulated ubiquitination. *β*‐Trcp is the main E3 ubiquitin ligase that facilitates I*κ*B ubiquitination and subsequent NF‐*κ*B activation 81. In general, the expression level of *β*‐Trcp is significantly up‐regulated in melanoma cells compared with that in melanocytes. Overexpression of BRAF in melanocytes potentiated *β*‐Trcp expression and NF‐*κ*B activity, while inhibition of either BRAF or downstream MEK led to the reduction in *β*‐Trcp expression and NF‐*κ*B activity, proving that the hyperactivated BRAF‐MEK signaling cascade in melanoma sustained NF‐*κ*B activation through the up‐regulation of *β*‐Trcp expression 82. This study illustrates the critical role of major genetic event in constitutive NF‐*κ*B activation via *β*‐Trcp mediated‐ubiquitination modification (Fig. 2). Moreover, *β*‐catenin, another target of *β*‐Trcp, is also a tumor facilitator in melanoma. In tumorigenesis, *β*‐catenin worked as a transcriptional factor in the Wnt/*β*‐catenin/T‐cell factor (Tcf) signaling pathway. Normally, glycogen synthase kinase 3*β* (GSK3*β*) is associated with the phosphorylation of *β*‐catenin, which results in the degradation of *β*‐catenin via the *β*‐Trcp mediated ubiquitin‐proteasome pathway. However, when GSK3*β*‐induced phosphorylation of *β*‐catenin was inhibited, the interaction between *β*‐catenin with *β*‐Trcp will be blocked, followed by impaired *β*‐catenin ubiquitination and degradation 83.

The activation of NF‐*κ*B in melanoma is not only determined by *β*‐Trcp, but also related to other upstream regulators. Receptor‐binding protein kinase (RIP1) is the one with integrated influence on both cell survival and cell death. Upon TNF*α*R1 stimulation, RIP1 forms a complex with TRADD, TRAF2, cIAP1, and cIAP2, and results in stabilization of RIP1 through K63‐linked polyubiquitination and/or linear ubiquitination of the protein carried out by TRAF2/cIAPs and linear ubiquitin chain assembly complex (LUBAC), respectively. Then, the ubiquitinated RIP1 binds to the TAB 2/TAB 3/TAK1 complex and NEMO, leading to activation of NF‐*κ*B 84, 85, 86, 87. In a recent study, Liuet al. found that RIP1 was frequently up‐regulated in human melanoma and played an oncogenic role, and RIP1 promoted melanoma cell proliferation through the activation of NF‐*κ*B. Notably, the ubiquitination of RIP1 was also prominently increased in melanoma cells, which was responsible for the high expression of RIP1 and constitutive activation of NF‐*κ*B. Through the blockage of TNF*α*R1 stimulation and RIP1 ubiquitination, the activity of NF‐*κ*B and the growth of melanoma were impeded 88.

### Regulation of MITF by ubiquitination and SUMOylation

MITF is a transcription factor ubiquitously expressed in different types of cells, especially playing an important role in the development and function of neural crest‐derived melanocytes and optic neuroepithelium‐derived retinal pigment epithelium cells 89. The amplification of MITF frequently occurs in melanoma, which results in the transcription of numerous oncogenic genes and tumor growth 14, 90, 91. Except the transcriptional modification, the expression and activity of MITF is regulated by multiple posttranscriptional approaches, including ubiquitination and SUMOylation.

In 2000, through the yeast two‐hybrid system and GST pull‐down assays, Xu et al. found that the ubiquitin‐conjugating enzyme hUBC9 directly interacted with MITF and subjected it for degradation by ubiquitin‐proteasome system. The serine73 phosphorylation site was essential for MITF ubiquitination and hUBC9‐dependent degradation, since the serine to alanine mutation at amino acid 73 (S73A) almost completely abrogated MITF ubiquitination. Further, lysine 201 was identified as the potential ubiquitination site, for lysine to arginine mutation (K201R) prevented the degradation of MITF by hUBC9 92. Since protein phosphorylation may lead to a conformational alteration that exposes regions required for proteolysis, phosphorylation on S73 may favor hUBC9 association and targets MITF protein for ubiquitination on K201. Nevertheless, the subsequent researches have revealed that MITF is subject to SUMO modification, which is strongly associated with hUBC9. In vitro study indicates that SUMOylation of MITF critically depends on E2 conjugating enzyme hUBC9 93, 94. Therefore, it is more likely that hUBC9 is involved in SUMOylation rather than ubiquitination of MITF. Additional studies will be needed to provide an explanation for this discrepancy. Later on, through a shRNA library against DUBs, USP13 was screened out as the deubiquitinase of MITF. Overexpression of USP13 in melanoma cells prolonged the half‐time of MITF expression in an enzymatic‐dependent manner. After the knockdown of USP13, the mRNA level of MITF downstream target gene *Trpm1* and the activity of Trpm1 promoter‐driven luciferase reporter was significantly dampened, suggesting that the USP13 regulated MITF target genes by influencing MITF binding to its target promoters. More importantly, USP13 deficiency resulted in impaired melanoma growth both in vitro and in vivo, and reintroduction of MITF reversed the inhibitory effect of USP13, demonstrating that MITF contributed to melanoma progression under the ubiquitination modification of USP13 95.

MITF is additionally modified by ubiquitin‐like proteins in mammalian cells. Through the immunoprecipitation analysis, Murakami *et al*. first found the direct interaction between MITF and SUMO1/SUMO2. The point mutation analysis showed that lysine 182 and lysine 316 are the two sites essential for MITF's SUMO‐conjugation both in vitro and in vivo. Furthermore, the SUMOylation‐resistant MITF led to the increase of transcriptional activity on some but not other MITF‐responsive promoters, implying that MITF's transcriptional output is subtly regulated by the extent of SUMOylation 93. Recently, a germline missense substitution in MITF (Mi‐E318K) was identified to occur at a significantly higher frequency in melanoma patients. The Mi‐E318K mutation severely impaired the SUMOylation of MITF and augmented the MITF protein binding to the HIF1A promoter and increased its transcriptional activity. Moreover, melanoma cells stably expressing Mi‐E318K showed enhanced migratory, invasive and clonogenic capacity, but little alteration of proliferation, which were reminiscent of melanoma‐initiating cells with increased invasive and division potential but with a slow growth rate 96. Therefore, SUMOylation maybe greatly implicated in melanoma onset and progression (Fig. 2).

### Cell cycle regulators and ubiquitination

Uncontrolled cell cycle progression is one of the hallmarks of cancer, including melanoma 97. Early genome‐wide association studies on familial melanoma identified that cyclin‐dependent kinase inhibitor 2A (*CDKN2A*) was a melanoma risk loci, which encodes two tumor suppressors, *P14* ^*ARF*^and *P16* ^*INK4a*^. Loss of p16^INK4a^ abrogates the inhibition of cyclin‐dependent kinase 4/6 (CDK4/6)–cyclin D pathway, and then cooperates with hyperactivated RAS‐RAF to induce melanoma 98, 99, 100, 101. Therefore, intervention on cyclin D and other cell cycle regulators is feasible in restraining melanoma progression. Notably, up to 20% of human melanomas with cyclin D1 overexpression lack genetic perturbations at the *CCND1 *locus 102. Thus, the dysregulation of cyclin D1, as well as other cell cycle regulators, is attributed to alternative mechanisms, including ubiquitination.

FBXO31 is a member of F‐box family located at chromosome 16q24.3 with the frequent loss of heterozygosity in different kinds of cancers 103, 104, 105. A previous study revealed that ectopic expression of FBXO31 in melanoma cells led to G1 arrest paralleled with remarkable reduction in cyclin D expression. The FBXO31‐mediated cyclin D1 degradation did not occur during the transcriptional period but instead in the posttranslational period, and the down‐regulation of cyclin D caused by FBXO31 occurred through the proteasomal pathway. Further, the coimmunoprecipitation analysis showed the direct interaction between FBXO31 and cyclin D, and FBXO31 was responsible for the ubiquitination of cyclin D 106. Recently, the E3 ubiquitin ligase FBXO4 was also implicated in regulating cyclin D expression and cell cycle progression. Using a transgenic melanoma mouse model, Lee et al. proved that FBXO4 deficiency induces melanoma in BRAF‐activated mice, with the expression of cyclin D accumulated in the nucleus of melanoma cells in the presence of FBXO4 inactivation. Moreover, the FBXO4 I377M mutant in which isoleucine 377 is replaced by methionine was identified to occur at a frequency of 8%, and led to impaired cyclin D1 recruitment and subsequent ubiquitination. The function of this mutation seems to be specific for cyclin D1, for FBXO4 I377M is still capable of regulating Trf1, another known substrate of FBXO4. Thus, these findings reveal a tumor suppressive role of FBXO4 in melanoma and provide novel insights into cyclin D1 ubiquitination modification 107.

During the cell cycle progression, cyclin‐dependent kinase (CDK) inhibitor p27^Kip1^ inhibits the kinase activity of G1‐cyclin‐CDK complexes and negatively regulates cell cycle from G1 to S phase 108, 109. Low levels of p27^Kip1^ expression are associated with poor prognosis in melanoma. Remarkably, the altered expression of p27^Kip1^ during the cell cycle does not occur in the mRNA level, but in the protein level through ubiquitination‐mediated degradation in the late G1 phase 110. The F‐box protein Skp2, which forms a SCF^Skp2^‐ubiquitin ligase complex, is a specific E3 ligase for p27^Kip1^. In melanoma cell lines, the expression of Skp2 is significantly increased and in a negative correlation with p27^Kip1^. Moreover, knockdown of Skp2 led to the accumulation of p27^Kip1^ and impaired tumorigenicity in melanoma cells, implying the significant role it plays in ubiquitin ligase activity of Skp2 in melanoma growth and cell cycle progression (Fig. 2) 111. Pivotally, Skp2 is localized in both nucleus and cytoplasm. Through the immunohistochemistry staining analysis, previous studies have shown that both the nuclear and cytoplasmic skp2 expressions were increased during melanoma progression, and the cytoplasmic skp2 expression was highly associated with melanoma patient survival 112, 113. Moreover, Qu et al. found that the total amount of Skp2 was also remarkably increased as melanoma progressed 114. In melanoma cell lines, the expression of Skp2 is significantly up‐regulated than normal human melanocyte and in a negative correlation with p27^Kip1^ 3, 111. The knockdown of Skp2 led to the accumulation of p27^Kip1^ and the impaired proliferation of melanoma cells in vitro. More importantly, inhibition of Skp2 resulted in suppressed xenograft tumor growth and increased cell apoptosis in nude mice in vivo 111.Collectively, these studies revealed not only the great value of Skp2 in predicting melanoma progression and prognosis, but also the potential of targeting Skp2 in melanoma therapy.

## Ubiquitination in Immune System and Melanoma Pathogenesis

The importance of immune responses in melanoma pathogenesis has long been appreciated. Dysregulated tumor‐associated T cells, natural killer cells, and macrophages are key components of immunologic contributors of melanoma progression 115, 116, 117. Recently, ubiquitination has been implicated in regulating the functions of various immune cells and the crosstalk between immune system and ubiquitination opened up a brand new era for the understanding of cancer. Thus, intervention of ubiquitination is not only meaningful for melanoma cell itself, but also of great importance in melanoma‐associated immunologic factors.

In 2014, through analyses of the BioGPS database, Zou et al. found that USP15 was abundantly expressed in immune cells, and the USP15 deficiency promoted the TCR^+^CD28^−^stimulated production of cytokines, such as interleukin 2 (IL‐2) and interferon‐*γ* in naive CD4^+^ T cells. In response to Listeria infection, the *Usp15* ^−/−^ T‐cell reconstituted hosts had reduced bacterial load in the liver and increased survival rate, implying that USP15 is dispensable for T‐cell function under infectious challenges. Specifically, USP15 stabilized an E3 ubiquitin ligase, MDM2, which in turn negatively regulated T‐cell activation by targeting the degradation of the master transcriptional factor of T‐cell activation, NFATc2. Then, the role of USP15 in regulating antitumor host defences was proved in a B16 melanoma model. B16‐challenged Usp15^−/−^ mice had an increased frequency of IFN‐*γ* ^+^ CD4^+^ T cells infiltrating to the tumors, as well as a profound reduction in B16 tumors size and tumor‐induced lethality 118. Therefore, targeting USP15 could be a valuable strategy for restraining melanoma growth by activating antitumor immunity.

Smad4 is another master transcriptional factor of T‐cell activation. In 2013, Yoon et al. reported that B16 melanoma growth and LN metastases were prominently suppressed in T‐cell‐specific *Smad4* knockout mice. In line with this, CD8^+^T‐cell infiltration was remarkable in the melanomas of *Smad4* ^−/−^mice, while it was absent in those of *Smad4* ^+/+^ mice, suggesting that the inhibition of Smad4 signaling could potentiate antitumor immunity. In addition, the employment of activin receptor‐like kinase5 (ALK‐5) inhibitor EW‐7197 induced ubiquitin‐mediated degradation of Smad4 and subsequently up‐regulation of eomesodermin in CD8^+^ T cells of melanoma‐bearing mice, thus enhancing the cytotoxic effect of T cells and leading to the regression of melanoma growth. The Smad4 signaling is a promising target for ubiquitination modification in melanoma‐associated T cells 117.

Aside from adaptive immune cells, innate immune cells are also implicated in the antitumor immunity, including NK cells. The activation of NK cells is initiated by the engagement of the NK receptor with glycolipid antigens such as *α*‐galactosylceramide (*α*‐GalCer), an agonistic ligand for NK cells presented by the MHC class I‐like molecule CD1d, which results in the production of cytokines such as IFN‐*γ* and IL‐4. However, injection of *α*‐GalCer into mice results in the hypo‐ or unresponsiveness of NK cells to restimulation, which is called NK cell anergy induction and limited the efficacy of activating NK cells in killing tumor cells. Cbl‐b is an E3 ligase which recruits both E2‐ubiquitin complex via its RING domain and the substrate through its protein‐interacting domains. Recently, the up‐regulation of Cbl‐b was implicated in NK cell anergy induction. Cbl‐b‐promoting monoubiquitination of CARMA1, a critical signaling molecule in NF‐*κ*B activation, disrupts its complex formation with Bcl10, which in turn leads to NK‐T cells' anergy induction. Cbl‐b deficiency prominently rescued the decreased IFN‐*γ* production and failed melanoma rejection observed in inactivated NK cells 119.In addition, genetic deletion of the Cbl‐b or targeted inactivation of its E3 ligase activity licensed NK cells to spontaneously reject melanoma metastasis through the ubiquitination of Tyro3, Axl, and Mer, which are members of TAM tyrosine kinase receptors. More importantly, administration of low‐dose warfarin, which inhibits TAM receptor activity in vivo, markedly reduced melanoma metastases to the lungs and distal organs in *Cbl‐b* ^+/+^ and *Cbl‐b* ^+/−^mice but had no apparent effect on *Cbl‐b*‐defective mice. In addition, warfarin treatment resulted in enhanced in vivo NK cell cytotoxicity and the absence of NK cells abolished the effects of warfarin on metastatic melanomas. This study demonstrates the antimetastatic activity of warfarin via Cbl‐b and TAM tyrosine kinase receptors in NK cells, revealing that targeting Cbl‐b by genetic or pharmacologic approaches may be a valuable strategy for awakening the innate immune system to kill cancer metastases 115.

## Targeting Ubiquitination in Melanoma

In melanoma development, tumor cells tend to drive the ubiquitin‐proteasome system to accelerate the degradation of tumor suppressor proteins (such as p27^Kip1^) and promote the abnormal stabilization of oncogenic proteins (such as NF‐*κ*B) in order to facilitate cell growth and survival 120.

The first proteasome inhibitor approved by FDA is bortezomib, a member of general proteasome inhibitor which was originally used for the treatment of multiple myeloma 96. Later, this drug was found to be associated with global inhibition of protein degradation and normal cell toxicity, which led to the study being discontinued in other cancer researches, including melanoma 121, 122. Subsequently, drugs with better specificity targeting enzymes upstream of the proteasome such as E3 ubiquitin ligase became more attractive. MDM2 is an E3 ubiquitin ligase with the ability to regulate tumor suppressor P53 and potentiate Notch signaling by degrading Numb 122, 123. Nutlin‐3a, a member of imidazoline compounds, has been widely reported as a MDM2 inhibitor and revealed the suppressive effect of melanoma and other cancers including leukemia, retinoblastoma, and neuroblastoma 124. Nutlin‐3a can potently bind to the hydrophobic cleft in the N‐terminus of MDM2, preventing its association with P53 122, 123. Now the pharmacologically optimized form of Nutlin‐3, RG7112, has completed phase I clinical trials for the treatment of both solid tumors and hematologic neoplasms. However, one of the phase I clinical trials in patients with liposarcoma revealed modest effect, with one partial response and stable disease in 70% of the cohort. However, serious adverse events occurred in 40% of the patients. In addition, 22% of patients with relapsed/refractory leukemia who experienced RG7112 in phase I trials underwent grade 3 and 4 febrile neutropenia 125. Moreover, an MDM2 homolog, MDMX, which is up‐regulated in approximately 65% human melanomas, can bind with the C‐terminal of MDM2 to form MDM2‐MDMX heterodimer that enhances the ubiquitination and degradation of P53 126, 127. In 2013, a molecule named ATSP‐7041 was reported of the antitumor potential by potently inhibiting both MDM2 and MDMX and preserving the function of wide‐type P53 127.

The ubiquitin‐like protein NEDD8 modulates the activity of Cullin‐RING ubiquitin ligase (CRLs), one of the major class of plant E3 ubiquitin ligases. CRLs covalently bind to their cullin subunits 128. In the process of NEDDylation, NEDD8 is activated by an E1‐like ubiquitin‐activating enzyme named NEDD8‐activating enzyme (NAE) and conjugated to Ubc12 and Nce2 by an analogous ubiquitination process in an ATP‐dependent manner 129. Previous studies revealed that NEDDylation was increased in melanoma cell lines and tissues, suggesting its prodevelopment role in melanoma 122. Recently, a NAE inhibitor named Pevonedistat (MLN4924) finished its study in phase I clinical trial of melanoma treatment. MLN4924 is a synthesized derivative of N6‐benzyl adenosine which structurally resembles adenosine 5′‐monophosphate (AMP) 130, 131. In melanoma, MLN4924 competitively inhibits NAE activity and prevents NEDDylation, resulting in the stabilization of proteins with cullin such as MLX, EID1, and MAGEA6 131. However, NAE is not the only target of MLN4924. Cdt1, which mediates DNA replication and accumulates in S phase of cell, and promotes the cancer cell death, is stabilized by MLN4924 through blocking the function of CRL1‐Skp2 and the formation of Cul4‐Rbx1‐Ddb1‐Cdt2 complex 129.During a phase I trial of MLN4924 in melanoma, 37 patients received different dosages of MLN4924. One patient had partial response and 15 patients achieved stable condition. However, two of them underwent acute organ failure, one patient developed myocarditis, acute renal failure, and hyperbilirubinemia simultaneously 132.

Siah2 is a RING finger E3 ubiquitin ligase and an important regulator of hypoxia‐activated pathways 133. It can be stabilized by the deubiquitination modification of USP13 and regulates Ras/Raf signaling pathway 134, 135. Since both hypoxia and Ras/MAPK play significant roles in melanoma development, siah2 is recognized as a potential drug target in melanoma. Vitamin K3 (menadione, MEN) has been extensively studied as an oxidative stress‐inducing quinone that causes cytotoxicity through increasing peroxidase production or consuming intracellular glutathione 135. It was indicated that MEN could bind with Siah2 on Ser39 of its substrate‐binding domain (SBD) to attenuate its self‐ubiquitination, leading to the increased expression of siah2 substrates PHD3 and Sprouty2, which subsequently attenuated the expression levels of HIF‐1a and pERK, and block melanoma tumorigenesis 136.

Another significant drug target of melanoma is deubiquitinase. USP9X is demonstrated as a protector of anti‐apoptotic protein Mcl‐1, preventing its ubiquitination and degradation 137. Benzyl ITC (BITC) and phenethyl ITC (PEITC) belong to the isothiocyanates (ITCs) class, both of which can increase the ubiquitination of Mcl‐1 through targeting USP9X and attenuate the cells' viability 138, 139. ITCs have been widely studied in many cancers including lung cancer, oral cancer, and leukemia 138, 140, 141. In oral cancer and lung cancer, PEITC is under the phase II clinical trials, and in melanoma and leukemia, PEITC is under the phase I trials.

In addition, it is of high possibility that these drugs not only impact the tumor itself, but also play crucial roles in modulating tumor‐associated immunity. As previously mentioned, Nutlin‐3a can efficiently bind to the hydrophobic cleft and inhibit the activity of MDM2. Thus, Nutlin‐3a may suppress tumor growth by potentiating the function of CD4^+^T cells. Moreover, it was reported that MLN4924 could impact the proliferation and cytokine production of T cells in response to *α*‐CD3/CD28 stimulation 142, 143. When stimulated with lower doses of *α*‐CD3, MLN4924 treatment can result in increased TCR‐stimulated cytokine production, proliferation, and iTreg development in both T cell lines and purified primary T cells. However, under high doses of *α*‐CD3/CD28 stimulation, MLN4924 tends to impair the proliferation, differentiation, and cytokine (such as IL‐2) production of the CD4^+^T cells 142, 143. It has been revealed that MLN4924 modulated T cells proliferation and cytokine production through the suppression of CRL activity and the reduction of Ubc12 expression 143, 144. Based on this, we speculate that the MLN4924‐mediated T cells proliferation and cytokine production is dependent on microenvironment, and may subsequently impact the killing effect of T cells on melanoma cells. Furthermore, vitamin K was once reported as an activator of NK cells. In 2005, Tremante et al.^145^ found that the combination of *α*‐tocopheryl succinate, vitamin K3, and vitamin C could significantly up‐regulate activating NK cell ligands, including the ligands of natural cytotoxicity receptors of melanoma cells, which subsequently increased the NK cell‐mediated lysis and melanoma cell death.

## Concluding Remarks

Ubiquitination and deubiquitination influence diverse cellular biological activities and impact the development of various tumors, including melanoma. Mutations of several ubiquitination‐associated enzymes, such as *BAP1*,* FBXW7* and *PARK2*, can lead to melanoma tumorigenesis. While *BRAF* and *NRAS* mutation are the most two common mutations in cutaneous melanoma, *BAP1* mutation frequently occurs in uveal melanomas exclusively with *BRAF* or *NRAS* mutations 146, suggesting that *BAP1* mutation may be a specific genetic marker for uveal melanoma. Moreover, *PARK2* gene is frequently mutated in melanoma cell lines or tumors harboring either *BRAF* or *RAS* mutation 75, which may contribute to the accuracy of genetic diagnosis of melanoma patient.

The tumor‐associated T cells and natural killer cells are two key immunologic components of melanoma progression. Nevertheless, the mechanism underlying the regulation of these immune cells by ubiquitination‐associated enzymes remains unclear. A major challenge in future studies is to identify the relationships between ubiquitin ligases and their substrates in those immune cells. Equally important is to uncover the upstream regulators of the ubiquitin ligases and the mediated pathways in immune cells.

We have also introduced several drugs targeting the ubiquitin‐proteasome system. All of which need further study to evaluate their efficacy and side effect. Given the complexity of the ubiquitin‐associated enzymes‐substrates network, drugs targeting conjugation enzymes‐ubiquitin receptors interactions requires higher selectivity. Although targeting the ubiquitination is promising, combining with individualized diagnosis and treatment may be more useful for melanoma treatment.

## Conflict of Interest

None declared.
